# Phenylalanine Butyramide Is a New Cosmetic Ingredient with Soothing and Anti-Reddening Potential

**DOI:** 10.3390/molecules26216611

**Published:** 2021-10-31

**Authors:** Ritamaria di Lorenzo, Antonietta Bernardi, Lucia Grumetto, Antonia Sacchi, Carmen Avagliano, Serena Coppola, Anna Fiorenza de Giovanni di Santa Severina, Cristina Bruno, Lorella Paparo, Sonia Laneri, Irene Dini

**Affiliations:** 1 Department of Pharmacy, University of Naples Federico II, Via Domenico Montesano 49, 80131 Napoli, Italy; ritamaria.dilorenzo@unina.it (R.d.L.); antonietta.bernardi@unina.it (A.B.); lucia.grumetto@unina.it (L.G.); antonia.sacchi@unina.it (A.S.); carmen.avagliano@unina.it (C.A.); 2 Department of Translational Medical Science, University of Naples Federico II, Via Sergio Pansini 5, 80131 Naples, Italy; serenacoppola@gmail.com (S.C.); fiorenzadegiovanni@gmail.com (A.F.d.G.d.S.S.); cristina.bruno@unina.it (C.B.); paparolorella@gmail.com (L.P.); 3 ImmunoNutritionLab at the CEINGE-Biotecnologie Avanzate s.c.ar.l Research Center, University of Naples Federico II, 80131 Naples, Italy

**Keywords:** butyrate, skin microbiota, skin permeation, erythema index, soothing and anti-reddening effect

## Abstract

Human skin is colonized by diverse commensal microbes, making up the skin microbiota (SM), contributing to skin integrity and homeostasis. Many of the beneficial effects aroused by the SM are exerted by microbial metabolites such as short-chain fatty acids (SCFAs), including butyric acid. The SCFAs can be used in cosmetic formulations against skin diseases to protect SM by preserving and/or restoring their natural balance. Unpleasant sensorial properties and unfavorable physico-chemical properties of butyrate strongly limit its cosmetic use. In contrast, some butyrate derivatives, including phenylalanine butyramide (C_13_H_18_N_2_O_2_, FBA), a solid form of butyric acid, are odorless while retaining the pharmacokinetic properties and safety profile of butyric acid. This study assessed the FBA’s permeation across the skin and its soothing and anti-reddening potential to estimate its cosmetic application. The dosage method used to estimate FBA’s levels was validated to be sure of analytical results. The FBA diffusion tests were estimated in vitro using a Franz-type vertical diffusion cell. The soothing action was evaluated in vivo by Colorimeter CL400, measuring the erythema index. The results suggest that the FBA represents an innovative way to exploit the benefits of butyric acid in the cosmetic fields since it cannot reach the bloodstream, is odorless, and has a significative soothing action (decrease the erythema index −15.7% after 30′, and −17.8% after 60′).

## 1. Introduction

The skin is the most significant interface between the human body and the environment. Exposure to UV B (UVB, wavelength of 280–320 nm) can generate reactive oxygen species (ROS) in the skin, exposing the biological tissue to oxidative stress [1], lipid peroxidation [2], chronic inflammation [3], and DNA damage [4,5,6], which can result in erythema, edema, and epidermal hyperplasia [7]. The skin is colonized by diverse commensal microbes composed of bacteria, fungi, viruses, archaea, and mites as a part of a network that is defined as SM that mediates essential physiological and pathological processes [8] and ensures its homeostasis contributing to the skin barrier function [9,10]. The skin microbes release enzymes involved in the stratum corneum renewal and desquamation process (proteases), lipidic film surface breakdown (lipases), and able to degrade urea (ureases). Moreover, the microbiota produces bacteriocins, biofilms [11,12], antimicrobial peptides [13,14], and indoles that inhibit many molds and yeasts [15]. The microbial metabolites (i.e., short-chain fatty acids including butyric acid) are responsible for some beneficial effects produced by the SM, including the dysbiosis (imbalances in the SM composition) enhancement due to pathological (i.e., dermatological diseases) and non-pathological (i.e., aging) skin conditions [16]. The fatty acids, secreted by the sebaceous glands, acidify the skin’s pH (it varies between 4.2 and 7.9) [17] and improve the epithelial barrier function by decreasing permeability, eliciting the eutrophic effect on the skin, and suppressing cutaneous inflammatory reactions [9,18]. Therefore, SCFAs could represent a novel strategy in cosmetic formulations for skincare. Previous studies have shown the significant role of butyric acid, the major metabolite made by *Staphylococcus epidermidis* fermentation, in inflammatory skin diseases [19]. It interacts with receptors, which secrete inflammatory cytokines expressed by keratinocytes [19]. Unfortunately, unfavorable sensorial and physicochemical properties strongly limit the dermatologic use of butyric acid. Therefore, it was thought to synthesize synthetic analogs to allow its use in the dermatological and cosmetic fields. The phenylalanine butyramide (FBA) proved noteworthy as a valid alternative for the topical use of butyrate among the synthesized derivatives. It releases butyric acid very quickly, has no smell, and has the same pharmacokinetic and safety profiles as butyric acid [20]. It doesn’t show any genotoxicity in vitro. The Ames test proved the absence of mutagenic properties. The absence of chromosomal breaks was showed by the micronucleus test [20].

In this study, the cosmetic potential of the FBA was evaluated. The potential permeation through the skin and the presence in the bloodstream were evaluated by in vitro experiments. The soothing and anti-reddening effects were proven by in vivo tests.

## 2. Results

**Figure 1:**
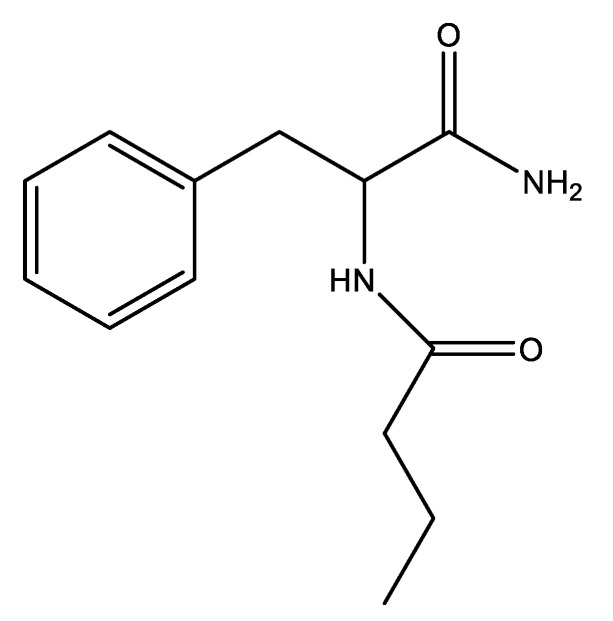
Phenylalanine butyramide (FBA).

FBA is a 1-carbamoyl-2-phenyl-ethyl derivative of butyric acid (Figure 1). In this study, its lipophilic potential was evaluated by in vitro studies validated by silicon studies.

### 2.1. In Vitro Studies

In vitro studies were performed to determine the FBA’s ability to permeate the skin and its soothing and anti-reddening potential. The FBA levels were evaluated after 1, 2, and 4 h.

#### 2.1.1. Partition Coefficient and In Silico Parameters

The lipophilicity of the FBA was evaluated by the determination of the octanol/water partition coefficient. The experimental Log P_o/w_, estimated by the shake-flask method, was 0.79 ± 0.12. The calculated Log P_o/w_ obtained by the XLOGP program was 0.79. Instead, the calculated Log P_o/w_ found by the ADMET^®^ program was 0.74. The value found experimentally was in line with those determined statistically. ADMET^®^ program calculated FBA Log Kp (skin permeation), resulting in 1919 cm/h.

#### 2.1.2. Determination of the FBA Levels and Skin Permeation

A new chromatographic method was used to determine the FBA concentration in the epidermis, dermis, and receptor compartment by time. The results of skin permeation were expressed in µg/mL and reported in Table 1.

The amount permeated in the skin was 0.2% at 60′ and 0.4% after 4 h.

**Figure 2:**
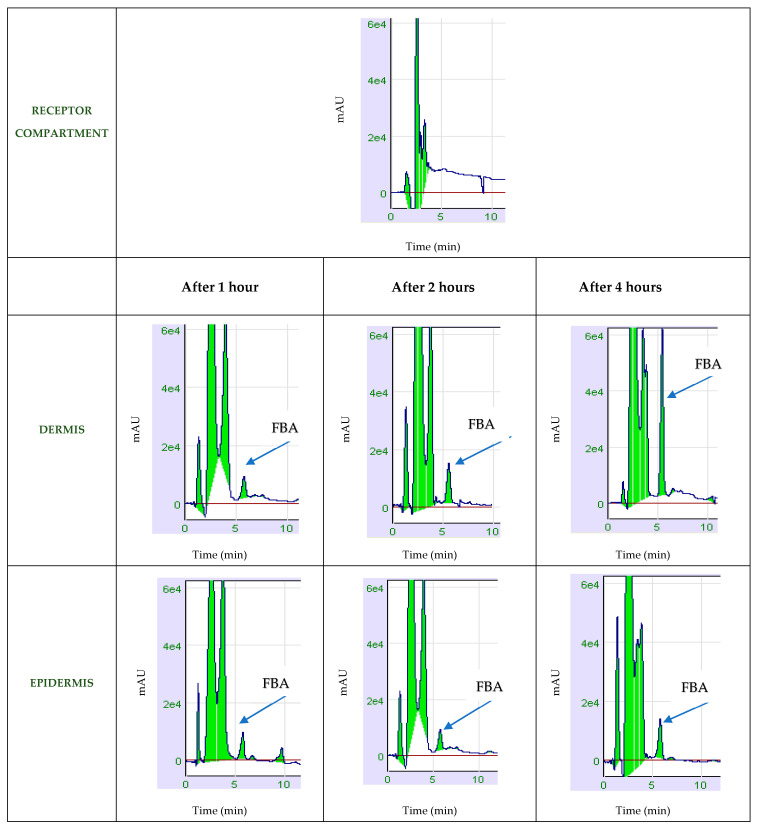
Chromatograms related to the determination of FBA levels in the skin.

The FBA chromatograms used to dosage the FBA in the epidermis and dermis are shown in Figure 2.

#### 2.1.3. Validation of the Analytical Method

The dosage method was validated to confirm the soundness of the results as required by the AOAC guideline [21]. Recovery was tested at different ultrasonication times. Ultrasonication time of 6 min was used for experimental purposes since it gave a reproducible average recovery of 95%. The matrix-matched calibration curve accomplished in the receptor phase supported a negligible (~95%) matrix effect, assessed as the ratio between the slopes of the calibration curve achieved in EtOH and measured in the receptor phase (saline solution, NaCl 0.9% *w*/*v*). The validation parameters are summarized in Table 2. Each concentration level used to determine the calibration curve was analyzed in triplicates.

The proposed method was sensitive and straightforward to quantify the FBA in skin layers at the end of a permeation experiment.

#### 2.1.4. Erythema Index

**Figure 3:**
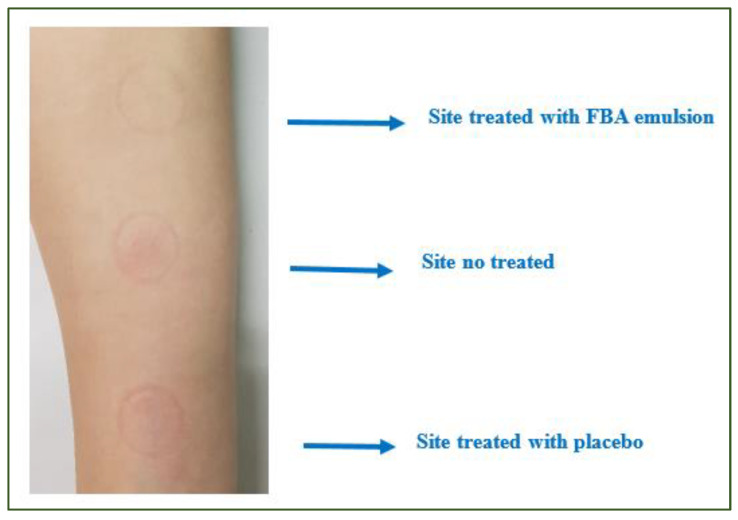
Image of the three test sites identified on the volunteer’s forearm at T1h.

The erythema index, calculated as skin redness (a*), was measured after 30 min and 1 h from the application of the W/O Emulsion containing FBA (A), the Placebo emulsion (P), and on the control site (Control). The results were reported in Table 3 and Figure 3.

There was a decrease in skin redness values on the control site (−8.6% after 30′ and −10.5% after 60′), and on the placebo site (−8.8% after 30′ and −11.8% after 60′). Moreover, a significative erythema index decrease (−15.7% after 30′ and −17.8% after 60′) was detected after applying the emulsion containing FBA (Table 3).

## 3. Discussion

Butyric acid has relevant anti-inflammatory, anti-redness, and lightening properties [20]. Nevertheless, the unpleasant organoleptic properties limit its use in the cosmetic field. Therefore, a butyrate (FBA) phenyl derivative, which can release butyric acid on the skin surface, was used to eliminate this unpleasant characteristic and test the potential cosmetic properties. The Regulation (EC) No 1223/2009 of the European Union [21,22] defines a cosmetic product as “any substance or mixture intended to be placed in contact with the external parts of the human body (epidermis, hair system, nails, lips, and external genital organs) or with the teeth and the mucous membranes of the oral cavity with a view exclusively or mainly to cleaning them, perfuming them, changing their appearance, protecting them, keeping them in good condition or correcting body odors” [22]. Therefore, a feature that differentiates the cosmetic from the pharmaceutical product is that the cosmetic product must not enter the bloodstream. Topically applied compounds can enter the body by diffusing the skin layers (transepidermal pathway) or through sweat ducts and hair follicles. In the first case, they can permeate the skin transcellular or intercellular. The solute’s molecular properties determine the access route. The lipophilic molecules prefer the intercellular route [23]. Therefore, the FBA’s lipophilicity was evaluated to establish which was the pathway of skin permeation. The substance’s amount permeated per unit area and unit time (J) depends on the skin’s permeability to the permeant (Kp) and the gradient of permeant concentration across the skin·(∆c). Kp depends on the partition coefficient P in the case of passive diffusion [24]. Previous studies evaluated skin permeation using in vitro models, including pig, rat, rabbit, mouse, and synthetic membranes [25,26].

In this study, the FBA log P value was evaluated using a shake-flask approach, and results were validated with two software to predict log P (XLOGP and ADMET). Similar to those determined statistically, the experimental results suggested that the FBA was a lipophilic substance. Thus, it was hypothesized that the transepidermal pathway of penetration was the most probable. Moreover, the low molecular weight and log P value made FBA a good candidate for skin application. Therefore, the FBA was applied at various times (1, 2, 4 h) on porcine skin, and its concentration was determined in the epidermis and dermis and the receptor compartment by a new validated chromatographic method. The selectivity, linearity, precision, and accuracy were evaluated for the method validation according to AOAC Guideline [21]. The selectivity was tested by modifying organic solvents to obtain FBA without interfering with peaks [27]. The recovery performed the extraction of both dermis and epidermidis at different ultrasonication times. The recovery of 95% was considered optimal for experimental purposes. The dosage method was validated by a correlation coefficient (r^2^) higher than 0.999, a sensitivity tested by measures of the LLOQ (lowest concentration of the calibration curve) and LOD (limit of detection) that can be quantified with accuracy (RE%) and precision (RSD%) < 20%, and an accuracy (RE%) lower than 15%.

The results of our study suggested that FBA was not able to reach the bloodstream so that the cosmetic properties could be attributable to butyric acid. Moreover, the low dosage of FBA in the skin implies that the FBA quickly transforms into butyric acid. In fact, as demonstrated in previous works [20], releasing butyrate from FBA occurs 2 h, so the concentration founded is lower than that at 1 h. After 4 h, the degraded quantity is constantly integrated into the epidermis by the donor compartment.

The FBA transformation into butyric acid could depend on enzymes (amidases) produced by the skin microbiome [28,29] or the skin’s pH. The FBA has two carboxylamine bonds: the first in the acid group of L-phenylalanine and the second between the amino group of phenylalanine and butyric acid. In an acidic environment, the amidases could break the amide bond and release the butyric acid. In vitro studies have shown that, in an acidic environment, FBA releases phenylalanine and butyric acid in a time-dependent manner [30]. Thus, it is plausible that at the acid skin’s pH, the FBA releases butyrate. Finally, the soothing and anti-reddening effects of an emulsion containing 1.5% FBA were tested in vivo in a skin area treated with Sodium Laureth Sulfate (SLES)-induced erythema and results compared to a placebo cream. This FBA concentration was chosen because the active ingredients are used in cosmetics at a variable concentration ranged from 1 to 2%. A significant decrease of the erythema index was detected after applying the emulsion containing FBA. The soothing and anti-reddening effects of the emulsion containing 1.5% FBA can be caused by butyrate since the FBA does not cross the skin, and previous studies have shown that butyrate regulates immune/inflammatory reactions in the skin [31] via binding to its receptor, GPR43 [19,32], which modulates the production of pro-inflammatory cytokines induced by skin injuries [32,33,34].

## 4. Materials and Methods

### 4.1. In Vitro Studies

#### 4.1.1. Reagent and Chemicals

Phenylalanine butyramide (FBA) (C_13_H_18_N_2_O_2_) (234.29 g/mol) (US patent US 2011 00983.19A1; 28 April 2011) was bought by ChiroBlock^®^ (Wolfen, Germany). Phosphate buffered saline (PBS) was provided by Sigma Aldrich (St. Louis, MO, USA). The analytical grade pure water (DW), acetonitrile (CAN), and ethanol (EtOH) were purchased from Exacta + Optech (S. Prospero, MO, ITALY).

#### 4.1.2. Determination of Octanol/Water Partition Coefficient

Octanol−water partition coefficients of FBA were determined using a shake-flask approach following the guidelines of the European Chemical Bureau [35]. Briefly, *n*-octanol−water mixtures were first vortexed for 1 min and then shaken overnight at room temperature (22 ± 2 °C) to reach the equilibrium and phase distribution. The phase ratio was water/*n* octanol 4/1. FBA was dissolved in a water-saturated organic solution at a concentration of 200 μg/mL. This solution (8 mL) was transferred to 15 mL centrifuge tubes and added to 2 mL of pre-saturated octanol. The tubes were shaken for one hour (a time window enough to reach equilibrium as demonstrated by preliminary experiments). After equilibration and phase separation, FBA was analyzed by liquid chromatography (LC) in both phases. An amount of 10 μL of the organic phase was sampled and added to 1 mL of mobile phase while the sampled aqueous phase was analyzed straight by liquid chromatography. The partition coefficient was calculated as the organic phase ratio and water concentration of FBA at equilibrium. Experiments were performed in triplicate.

#### 4.1.3. Validation of Octanol/Water Partition Coefficient

The XLOGP (Atomistic and knowledge-based method calculated by XLOGP program, version 3.2.2, courtesy of CCBG, Shanghai Institute of Organic Chemistry) and ADMET (Simulation Plus, West Lancaster, PA, USA) programs predicted the *n* octanol/water partition coefficient (log Poct/w). ADMET software result was given as log Kp*Kp (cm/h) defined below:

$$\mathrm{Kp}\;=\frac{\mathrm{Km}*D}{h}$$

Km is the distribution coefficient between stratum corneum and vehicle.

D is the average diffusion coefficient (cm^2^/h), and *h* is the skin thickness (cm).

#### 4.1.4. Tissue Preparation

For permeation analysis, porcine skin, coming from the outer part of the ear, was used. The skin was excised post-sacrifice from female and male animals (age 10–12 months, weight 150–190 kg) from a local slaughterhouse (Avellino, Italy) within 3 h from animal death. Subcutaneous fat was removed, and skin samples were maintained at 4 °C until the experiment was performed within 24 h. The skin was put on a filter paper (Fisherbrand™ Grade 601, Fisher Scientific, Leicestershire, UK) and cut into pieces of appropriate size before assembly in the Franz cells. The integrity of porcine skin was examined by measuring the impedance of the skin.

#### 4.1.5. Chromatographic Analysis for Skin Permeation Study

The quantitative determination of FBA was performed with a chromatographic system consisted of a high performance liquid chromatographic system (LC-20 AD VP; Shimadzu Corp., Kyoto, Japan), equipped with a 7725 Rheodyne injection valve fitted with a 20 μL loop, and an ultraviolet (UV)–visible detector (Shimadzu Model SPD10 AV) set at λ 200 nm. The chromatographic condition is reported in Table 4. The mobile phase solvents were vacuum-filtered through 0.45 μm nylon membranes (Millipore, Burlington, MA USA). Data acquisition and integration were accomplished by Cromatoplus 2011 software. Each sample was injected three times to test the instrument repeatability.

#### 4.1.6. Validation of FBA Extraction Method and HPLC Analysis

The AOAC guidelines were used to validate the method [21].

##### Selectivity

Blank samples of epidermis and dermis were blank. They were extracted at room temperature with 1 mL of EtOH. The solutions were analyzed to verify the presence of interfering peaks eluting at the retention time of FBA. The receptor solution of a blank permeation experiment was analyzed too. Blanks samples were spiked with known amounts of FBA (25, 10, and 5 µL of standard solution in ethanol 200 µg/mL). The dried specimens were extracted with 1 mL of EtOH in various conditions. The samples were centrifuged at 15.000 rpm for 10 min and sonicated for 6 min. The recovery of FBA from skin layers was assessed compared to the amount applied and evaluated by injection of spiked concentrations without skin layer samples.

The HPLC system was calibrated using standard synthetic solutions prepared by diluting the FBA in EtOH to four different concentration values ranging from 0.625 to 10 μg/mL. A stock solution of FBA with a 2 mg/mL concentration was prepared by dissolving 2 mg of FBA in 1 mL of ACN. Each concentration level was analyzed in triplicates. Linearity was assessed by inspecting the detection signals as a function of analyte concentrations by a regression line by the method of the least-squares.

Quantitative analyses were performed on matrix-matched calibration curves, peak area vs. concentration (μg/mL). The matrix-matched calibration curves were obtained from the analyses of blank sample extracts spiked FBA at the same range concentrations above reported.

##### Sensitivity

The analytical sensitivity defines the minor concentration of a measurand that an analytical procedure can reliably measure [36]. The lower limit of quantification (LLOQ) and the lower detection limit (LOD) explains this validation parameter.

- LLOQ

The method’s LLOQ was the concentrations of the analytes that provided signals equal to 10 times the noise signal of the analysis. It was evaluated on the synthetic standards and calculated on the standard deviation of the response (SDa) of the curve and the slope of the calibration curve (*S*). The standard deviation of the response was determined based on the standard deviation of *y*-intercepts of regression lines.

$$\mathrm{LOQ}\;=\;\frac{\mathrm{SDa}\;*\;10}{S}$$

- LOD

The method’s LOD was assumed as the concentrations of the analytes that provided signals equal to 3 times the noise signal of the analysis.

$$\mathrm{LOQ}\;=\;\frac{\mathrm{SDa}\;*\;3}{S}$$

##### Precision and Accuracy

The method’s precision was evaluated by running three replicate samples at four different concentration levels. The analyses were repeated along the two different days to cover both intra-day and inter-day precision.

The accuracy (relative error RE%) was measured as the difference between the theoretical amounts and the approximation and divided by the magnitude of the exact value. The precision (RSD%) was calculated as the coefficient of variation of the back-calculated concentrations.

##### Matrix Effect

Matrix effect was calculated as follows:

$$\mathrm{Matrix}\;\mathrm{effect}\;\left(\%\right)=\frac{\mathrm{Peak}\;\mathrm{area}\;\mathrm{of}\;\mathrm{analyte}\;\mathrm{in}\;a\;\mathrm{standard}\;\mathrm{solution}-\mathrm{peak}\;\mathrm{area}\;\mathrm{of}\;\mathrm{the}\;\mathrm{analyte}\;\mathrm{in}\;a\;\mathrm{sample}\;}{\mathrm{Peak}\;\mathrm{area}\;\mathrm{of}\;\mathrm{analyte}\;\mathrm{in}\;a\;\mathrm{standard}\;\mathrm{solution}}\%$$

All the analyses were expressed as the mean of the three analyses.

#### 4.1.7. Skin Permeation and Retention

The FBA diffusion tests were undertaken on Franz-type vertical diffusion cells (Ø 9 mm, 5-mL receptor compartment, SES GmbH-Analyse System, Bechenheim, Germany) with an effective diffusion area of 0.6 cm^2^ and receptor volume of 4 mL [37]. The total thickness of the skin was washed three times with 1 mL of water and mounted between the two halves of the cell with the stratum corneum facing the donor compartment. The receptor compartment was filled with 4 mL of degassed saline solution (NaCl 0.9% *w*/*v*), while in the donor compartment, was put 1 mL of a solution composed of 1 mg FBA dissolved in 1 mL of PBS/EtOH (70:30).

The Franz Cell system was maintained at a constant temperature of 37 ± 0.5 °C through thermostatic bath circulation, while the receptor medium was constantly kept under magnetic stirring through the experiments. At the end of the application time (1, 2, and 4 h), the skin was removed from the cell. Some aliquots were collected from the supernatant portion and analyzed by HPLC to quantify the amounts of FBA. The skin was wiped three times with paper soaked in distilled water, and the epidermis and dermis were separated. The skin was cut according to the permeation area (1 cm^2^) and heated at 50 °C with an air dryer for 30 s. Then epidermis was separated from the dermis by scraping with a scalpel. Experiments were performed in triplicate. Epidermis and dermis samples were placed in glass vials and extracted with 1 mL of EtOH. The samples were centrifuged at 15.000 rpm for 10 min and sonicated for 6 min. An amount of 1 mL of the samples was filtered using a 0.45 μm polyamide (PA) filter (Sartorius AG, Goettingen, Germany) and applied [37].

#### 4.1.8. Permeability Calculation

The skin permeability was calculated by dividing the amount of FBA present in the skin (epidermis and dermis) by skin surface area using the following formula:

$$D\left(\frac{µg}{cm}\right)=\frac{\mathrm{Amount}\;\mathrm{receptor}\;\mathrm{fluid}\;+\;\mathrm{Amount}\;\mathrm{skin}}{\mathrm{SSA}\;\left(\mathrm{skin}\;\mathrm{surface}\;\mathrm{area}\right)}$$

The ratio of the total amount of PP in the receptor fluid and skin to the amount of FBA applied was calculated to determine the total absorption rate:

$$Total\;absorption\;\left(\%\right)=\frac{\mathrm{Amount}\;\mathrm{receptor}\;\mathrm{fluid}\;+\;\mathrm{Amount}\;\mathrm{skin}}{\mathrm{Amount}\;\mathrm{total}}\times 100$$

### 4.2. In Vivo Study

The revisions recognized by the European Community of the principles of the Helsinki Declaration (1964) [38] and the Colipa Guidelines for evaluating the efficiency of cosmetic products (May 2008) [39] were followed to perform the experimental study.

#### 4.2.1. Study Design

An active substance with a 1.5% (1.5 × 10^4^ μg/mL) concentration (Emulsion with FBA) and a placebo cream were used to evaluate the FBA’s soothing action. A single-blind study was used to evaluate the soothing action of the FBA in W/O emulsion (Emulsion with FBA). The protocol of the Short Term Test provided the identification of three zones (4 cm^2^) on the fly part of the volunteers’ arm and the induction of the erythema using SLES at high concentration (5 mL of pure Sodium Laureth Sulfate at 30%) for 1 min, and successively gently cleaning with water and a tamponade tap dry with absorbent paper. The erythema’s area was divided into three portions: placebo, emulsion with FBA formulation, and control. The erythema index was quantified using the Colorimeter CL 400 (Courage + Khazaka electronic GmbH (Köln, Germany)) probe, which sends out white LED light, arranged circularly to illuminate the skin uniformly. The emitted light was scattered in all directions. Parts of it traveled through the layers, and some were scattered out of the skin. The light reflected from the skin was measured by the probe and expressed accordingly. The raw data of the probe were corrected with a particular color matrix as close as possible to typical values. The measured color of the skin was expressed as an XYZ-value (tristimulus) and was calculated into RGB values (red/green/blue) or L*a*b related value. The parameter a* (T30′–T60′) was measured after induction of erythema (T0) and after 30′ and 60′ from the application on the three areas. Instrumental measurements were carried out at T = 25 °C ± 2° and controlled humidity (45 ± 5% RH) was measured after an acclimatization period of about 30 min.

#### 4.2.2. Study Population

Twenty healthy female volunteers (caucasian ethnicity), from all social categories, aged 20–60 years, skin phototype: II-IV Fitzpatrick scale, who had no systemic pathologies, and who had not applied any product on the test site in the previous seven days, were enrolled. Excluded were pregnant or breastfeeding women, women with cutaneous hyper-reactivity or intolerance reactions to cosmetic products/ingredients, topical or systemic treatment with any drug that may interfere with the outcome of the test, women affected by skin diseases (eczema, psoriasis, lesions), topical use of retinoids in the previous six months at the start of the study or with systemic retinoids in the previous 12 months, and topical use of products based on alpha and beta-hydroxy acids in the 30 days before the start of the study.

The volunteers subscribed an informed consent to permit the process of the personal data and images taken during the in vivo tests for the uses permitted by D.Legs n. 196 of 30 June 2003, and in which they declared to know the composition of the active/formulation and no allergy to its components.

For each volunteer, a detailed form was prepared to use the samples provided by the R&D Cosmetics center of the Pharmacy Department.

#### 4.2.3. Cream Composition

The Emulsion with FBA contained two phases: phase O (oil phase) polyglyceryl-3 methylglucose 5.0%, cetyl alcohol 2%, and cetearyl alcohol 3%, and phase W (water phase) containing water (88.8%), FBA (1.5%), sodium benzoate 0.5%, potassium sorbate, and perfume (0.1%). The placebo cream contained all the ingredients without the FBA. The creams’ components were bought at ACEF (Fiorenzuola D’Arda, Italy) and Parfum by Farotti Essenze (Rimini, Italy). The two creams were made, homogenizing the two phases energetically at 70 °C using a Silverson L5M-A Laboratory Mixer (SBL, Shanghai, China), cooling in an ice bath, and adding the remaining components at room temperature. Finally, the pH (5.4–5.5) and viscosity (30.203–30.406 mPa; L4, 20 rpm) were tested using a Crison GPL20 pH-Meter (Crison, Barcelona, Spain) and a Visco Basic Plus rheometer (Fungilab, Barcelona, Spain).

#### 4.2.4. Statistical Analysis

Descriptive statistics were reported as means and standard deviations (SDs) for continuous variables. The independent sample *t*-test was performed to evaluate the differences among continuous variables. The level of significance for all statistical tests was two-sided, *p* < 0.05. All data were collected in a dedicated database and analyzed by a statistician using QSPR model implemented by Potts and Guy, 1992 Pharm. Res Swissadme^®^ (Swiss Institute of bioinformatics) [40] and ADME^®^ (Simulation Plus, West Lancaster, PA, USA) predictor software was used.

## 5. Conclusions

In this study, the skin permeation, and the soothing and anti-reddening effect of the FBA, a new odorless and rapidly absorbed by skin butyrate derivative able to release butyric acid in the skin were tested for the first time. The results suggest that FBA could not reach the bloodstream, and cosmetic benefits could be attributable to butyric acid. Therefore, the FBA represents an innovative way to exploit the benefits of butyric acid in the cosmetic field without the organoleptic limits of the latter.

## Figures and Tables

**Table 1:** FBA concentration in epidermis, dermis, and receptor compartment by time (µg/mL).

Time (h)	Conc. FBA in Epidermis (µg/mL)	Conc. FBA in Dermis (µg/mL) for PBs Donor	Conc. FBA in the Receptor Compartment (µg/mL)
1	1.49 ± 0.40	0.30 ± 0.07	0
2	0.37 ± 0.06	0.71 ± 0.11	0
4	1.73 ± 0.31	3.18 ± 0.49	0

**Table 2:** Validation parameters for the quantitative analysis of FBA.

Calibration Parameters	
Linear range (µg/mL)	0.5–10
Slope	13,178
Intercept	2195.5
r^2^	0.9995
Precision and Accuracy of the Chromatographic System	
Repeatability (n = 5); RSD (%)	4.80
Intermediate precision (n = 10); RSD (%)	8.70
LOQ (ng mL^−1^)	20,000
LOD (ng mL^−1^)	0.00991
Matrix effect	95.0

**Table 3:** Average of Erythema Index (a* mean values) at T0, T30′ and T60′ and average of decrease in erythema (%) after 30′ and 60′ for Emulsion with FBA (site A), Placebo (site P) and control site.

Site	Product Tested	a* Mean Values			Percentage Variations of Erythema Index	Percentage Variations of Erythema Index
		**T0**	**T30′**	**T60′**	**T30′ vs. T0**	**T60′ vs. T0**
A Area	Emulsion with FBA	9.63 ± 2.48	8.04 ± 1.63	7.63 ± 1.46	−15.7% *p* = 0.0013	−17.8% *p* = 0.0037
P Area	Placebo	9.68 ± 2.19	8.61 ± 1.36	8.32 ± 1.34	−8.58% *p* = 0.13	−10.53% *p* = 0.064
**Control site**	No treated	8.98 ± 2.26	8.04 ± 1.53	7.84 ± 1.41	−8.8% *p* = 0.071	−11.8% *p* = 0.024

**Table 4:** Chromatographic conditions.

Column	Phenyl Hexyl (250 × 4.6 mm, 100 Å) (Kinetex, USA)
Precolumn	4 × 3.0 mm; Phenomenex, CA, USA
Mobile phase	ACN:DW (30:70)
UV detection λ	200 nm
Flow rate	0.5 mL/min
Retention time	5.90 ± 0.5 min

## Data Availability

Data is contained within the article.

## Sample Availability

A sample of the compound is available from the authors.

## References

[1] Acker T., Fandrey J., Acker H. (2006). The good, the bad and the ugly in oxygen-sensing: ROS, cytochromes and prolyl-hydroxylases. Cardiovasc. Res..

[2] Biernacki M., Brzóska M.M., Markowska A., Gałażyn-Sidorczuk M., Cylwik B., Gęgotek A., Skrzydlewska E. (2021). Oxidative Stress and Its Consequences in the Blood of Rats Irradiated with UV: Protective Effect of Cannabidiol. Antioxidants.

[3] Dini I., Holban A.M., Grumezescu A.M. (2018). Spices and herbs as therapeutic foods. Food Quality: Balancing Health and Disease.

[4] Lopez-Camarillo C., Ocampo E.A., Casamichana M.L., Perez-Plasencia C., Alvarez-Sanchez E., Marchat L.A. (2012). Protein kinases and transcription factors activation in response to UV-radiation of skin: Implications for carcinogenesis. Int. J. Mol. Sci..

[5] Laneri S., Di Lorenzo R., Sacchi A., Dini I. (2019). Dosage of Bioactive Molecules in the Nutricosmeceutical Helix aspersa Muller Mucus and Formulation of New Cosmetic Cream with Moisturizing Effect. Nat. Prod. Commun..

[6] Dong K., Goyarts E., Rella A., Pelle E., Wong Y.H., Pernodet N. (2020). Age Associated Decrease of MT-1 Melatonin Receptor in Human Dermal Skin Fibroblasts Impairs Protection Against UV-Induced DNA Damage. Int. J. Mol. Sci..

[7] Dini I., Laneri S. (2021). The New Challenge of Green Cosmetics: Natural Food Ingredients for Cosmetic Formulations. Molecules.

[8] Boxberger M., Cenizo V., Cassir N., La Scola B. (2021). Challenges in exploring and manipulating the human skin microbiome. Microbiome..

[9] De Pessemier B., Grine L., Debaere M., Maes A., Paetzold B., Callewaert C. (2021). Gut-Skin Axis: Current Knowledge of the Interrelationship between Microbial Dysbiosis and Skin Conditions. Microorganisms.

[10] Belkaid Y., Tamoutounour S. (2016). The influence of skin microorganisms on cutaneous immunity. Nat. Rev. Immunol..

[11] Baldwin H.E., Bhatia N.D., Friedman A., Eng R.M., Seite S. (2017). The role of cutaneous microbiota harmony in maintaining a functional skin barrier. J. Drugs Dermatol..

[12] Williams M.R., Costa S.K., Zaramela L.S., Khalil S., Todd D.A., Winter H.L., Sanford J.A., O’Neill A.M., Liggins M.C., Nakatsuji T. (2019). Quorum sensing between bacterial species on the skin protects against epidermal injury in atopic dermatitis. Sci. Transl. Med..

[13] Nakatsuji T., Chen T.H., Narala S., Chun K.A., Two A.M., Yun T., Shafiq F., Kotol P.F., Bouslimani A., Melnik A.V. (2017). Antimicrobials from human skin commensal bacteria protect against *Staphylococcus aureus* and are deficient in atopic dermatitis. Sci. Transl. Med..

[14] O’Sullivan J.N., Rea M.C., O’Connor P.M., Hill C., Ross R.P. (2019). Human skin microbiota is a rich source of bacteriocin-producing staphylococci that kill human pathogens. FEMS Microbiol. Ecol..

[15] Gaitanis G., Tsiouri G., Spyridonos P., Stefos Τ., Stamatas G.N., Velegraki A., Bassukas I.D. (2019). Variation of cultured skin microbiota in mothers and their infants during the first year postpartum. Pediatr. Dermatol..

[16] Sfriso R., Egert M., Gempeler M., Voegeli R., Campiche R. (2020). Revealing the secret life of skin—With the microbiome you never walk alone. Int. J. Cosmet. Sci..

[17] Meisel J.S., Sfyroera G., Bartow-McKenney C., Gimblet C., Bugayev J., Horwinski J., Kim B., Brestoff J.R., Tyldsley A.S., Zheng Q. (2018). Commensal microbiota modulate gene expression in the skin. Microbiome.

[18] Dini I., Laneri S. (2021). Spices, Condiments, Extra Virgin Olive Oil and Aromas as Not Only Flavorings, but Precious Allies for Our Wellbeing. Antioxidants.

[19] Keshari S., Balasubramaniam A., Myagmardoloonjin B., Herr D.R., Negari I.P., Huang C.-M. (2019). Butyric Acid from Probiotic *Staphylococcus epidermidis* in the Skin Microbiome Down-Regulates the Ultraviolet-Induced Pro-Inflammatory IL-6 Cytokine via Short-Chain Fatty Acid Receptor. Int. J. Mol. Sci..

[20] Russo R., Santarcangelo C., Badolati N., Sommella E., De Filippis A., Dacrema M., Campiglia P., Stornaiuolo M., Daglia M. (2021). In vivo bioavailability and in vitro toxicological evaluation of the new butyric acid releaser N-(1-carbamoyl-2-phenyl-ethyl) butyramide. Biomed. Pharmacother..

[21] AOAC (2012). Appendix F: Guidelines for Standard Method Performance Requirements (SMPR).

[22] Consolidated Text: Regulation (EC) No 1223/2009 of the European Parliament and of the Council of 30 November 2009 on Cosmetic Products (Recast) (Text with EEA Relevance). https://eur-lex.europa.eu/legal-content/EN/TXT/?uri=CELEX%3A02009R1223-20210823.

[23] Ng K.W., Lau W.M. (2015). Skin Deep: The Basics of Human Skin Structure and Drug Penetration. Percutaneous Penetration Enhancers Chemical Methods in Penetration Enhancement.

[24] Sobańska A.W., Robertson J., Brzezińska E. (2021). Application of RP-18 TLC Retention Data to the Prediction of the Transdermal Absorption of Drugs. Pharmaceuticals.

[25] Todo H. (2017). Transdermal Permeation of Drugs in Various Animal Species. Pharmaceutics.

[26] Neupane R., Boddu S.H.S., Renukuntla J., Babu R.J., Tiwari A.K. (2020). Alternatives to Biological Skin in Permeation Studies: Current Trends and Possibilities. Pharmaceutics.

[27] Elias P.M. (1991). Epidermal barrier function: Intercellular lamellar lipid structures, origin, composition, and metabolism. J. Control. Release.

[28] Valente L.G., Pitton M., Fürholz M., Oberhaensli S., Bruggmann R., Leib S.L., Jakob S.M., Resch G., Que Y.-A., Cameron D.R. (2021). Isolation and characterization of bacteriophages from the human skin microbiome that infect *Staphylococcus epidermidis*. FEMS Microbes.

[29] Esgalhado M., Kemp J.A., Damasceno N.R., Fouque D., Mafra D. (2017). Short-chain fatty acids: A link between prebiotics and microbiota in chronic kidney disease. Future Microbiol..

[30] Schwarz A., Bruhs A., Schwarz T. (2017). The Short-Chain Fatty Acid Sodium Butyrate Functions as a Regulator of the Skin Immune System. J. Investig. Dermatol..

[31] Sujka W., Draczynski Z., Kolesinska B., Latanska I., Jastrzebski Z., Rybak Z., Zywicka B. (2019). Influence of porous dressings based on butyric-acetic chitin co-polymer on biological processes in vitro and in vivo. Materials.

[32] Karaki S., Mitsui R., Hayashi H., Kato I., Sugiya H., Iwanaga T., Furness J.B., Kuwahara A. (2006). Short-chain fatty acid receptor, GPR43, is expressed by enteroendocrine cells and mucosal mast cells in rat intestine. Cell Tissue Res..

[33] Traisaeng S., Herr D.R., Kao H.J., Chuang T.H., Huang C.M. (2019). A derivative of butyric acid, the fermentation metabolite of *Staphylococcus epidermidis*, inhibits the growth of a *Staphylococcus aureus* strain isolated from atopic dermatitis patients. Toxins.

[34] Krejner A., Bruhs A., Mrowietz U., Wehkamp U., Schwarz T., Schwarz A. (2018). Decreased expression of G-protein-coupled receptors GPR43 and GPR109a in psoriatic skin can be restored by topical application of sodium butyrate. Arch. Dermatol. Res..

[35] European-Chemical-Bureau, Dir 92/69/EEC. https://eur-lex.europa.eu/legal-content/EN/TXT/?uri=CELEX%3A31992L0069.

[36] Dini I., Graziani G., Fedele F.L., Sicari A., Vinale F., Castaldo L., Ritieni A. (2020). An Environmentally Friendly Practice Used in Olive Cultivation Capable of Increasing Commercial Interest in Waste Products from Oil Processing. Antioxidants.

[37] Padula C., Pappani A., Santi P. (2008). In vitro permeation of levothyroxine across the skin. Int. J. Pharm..

[38] Carlson R.V., Boyd K.M., Webb D.J. (2004). The revision of the Declaration of Helsinki: Past, present and future. Br. J. Clin. Pharmacol..

[39] Renner G., Audebert F., Burfeindt J., Calvet B., Caratas-Perifan M., Leal M.E., Gorni R., Long A., Meredith E., O’Sullivan Ú. (2017). Cosmetics Europe Guidelines on the Management of Undesirable Effects and Reporting of Serious Undesirable Effects from Cosmetics in the European Union. Cosmetics.

[40] Daina A., Michielin O., Zoete V. (2017). SwissADME: A free web tool to evaluate pharmacokinetics, drug-likeness and medicinal chemistry friendliness of small molecules. Sci. Rep..

